# High-Linearity High-Resolution Time-of-Flight Linear-Array Digital Image Sensor Using Time-Domain Feedback

**DOI:** 10.3390/s21020454

**Published:** 2021-01-11

**Authors:** Juyeong Kim, Keita Yasutomi, Keiichiro Kagawa, Shoji Kawahito

**Affiliations:** 1 Graduate School of Science and Technology, Shizuoka University, Hamamatsu, Shizuoka 432-8011, Japan; jkim@idl.rie.shizuoka.ac.jp (J.K.); kagawa@idl.rie.shizuoka.ac.jp (K.K.); 2 Research Institute of Electronics, Shizuoka University, Hamamatsu, Shizuoka 432-8011, Japan; kyasu@idl.rie.shizuoka.ac.jp

**Keywords:** CMOS image sensor (CIS), short-pulse, time-of-flight (ToF), indirect ToF, depth sensing

## Abstract

This paper presents a high-linearity high-resolution time-of-flight (ToF) linear-array digital image sensor using a time-domain negative feedback technique. A coarse ToF measurement loop uses a 5-bit digital-to-time converter (DTC) and a delayed gating-pulse generator for time-domain feedback to find the zero of the difference between ToF and the digital estimate of the gating-pulse delay while maintaining a constant operating point of the analog readout circuits. A fine ToF measurement uses a delta-sigma modulation (DSM) loop using the time-domain feedback with a bit-stream signal form. Because of the self-contained property of the DSM for low distortion and noise exploited by the oversampling signal processing, the proposed technique provides high-linearity and high-range resolution in the fine ToF measurement. A prototype ToF sensor of 16.8 × 16.8 μm^2^ two-tap pixels and fabricated in a 0.11 μm (1P4M) CMOS image sensors (CIS) process achieves +0.9%/−0.47% maximum nonlinearity error and a resolution of 0.24 mm (median) for the measurement range of 0–1.05 m. The ToF sensor produces an 11-bit fully digital output with a ToF measurement time of 22.4 ms.

## 1. Introduction

Among the various functions of CMOS image sensors (CISs), time-of-flight (ToF) range image sensors are receiving much attention for new markets and applications of CISs, including consumer, industrial, and scientific applications [1,2,3]. ToF range sensor applications require high precision, accuracy, linearity, and tolerance to ambient light. Direct-type ToF imagers using a single-photon avalanche diode, and all-digital-domain processing are excellent for high-accuracy ToF [4,5,6,7]. However, it requires complex hardware, specifically, if there is a need for extremely high-resolution and tolerance to ambient light. Indirect-type ToF imagers have an advantage of small pixel size, less circuit complexity, and relatively reliable range resolution, specifically for range measurements of a few meters. Two types of indirect ToF imaging methods exist, depending on the waveform of the modulated light. One is continuous wave (CW)-based [8,9,10,11,12,13,14,15], and the other is short pulse (SP)-based [16,17,18,19,20,21,22,23,24]. The SP-based indirect ToF has a better tolerance to ambient light because the light power is concentrated on the short pulse, and the charge-draining function of the ToF pixel reduces the influence of the ambient light. However, because of the analog-domain processing for ToF measurement, the SP-based indirect ToF sensor suffers from various analog imperfections, such as the nonlinearity of the pixel source-follower amplifier, the distortion of the waveform of the light pulse, finite photo-carrier response time in the photodiode, and the distortion of the gating pulse for demodulation. The full-well capacity of the pixel limits the range resolution or depth noise of indirect ToF sensors if the photon-shot noise is the liming factor of the range resolution [3]. Using a short pulse is effective for improving the range resolution. High-resolution ToF sensors designed with a pixel using a very short light pulse lower than 100 ps and a large full-well capacity larger than 1M electrons have range resolutions of sub-millimeter [25] and sub-100 micrometer [26]. Because of the short pulse, the nonlinearity and skew of the gating pulses become significant issues to be solved. Complicated off-line processing for nonlinearity correction and the on-chip skew calibration circuits are necessary for implementing a linear array ToF sensor.

To address to the issues of the SP-based indirect ToF sensors, this paper proposes an SP-based indirect ToF image sensor using the time-domain negative feedback technique. To the best of our knowledge, this work is the first attempt at a indirect ToF image sensor using a time-domain negative feedback technique. In the conventional indirect ToF image sensors, open-loop analog interface circuits and a successive analog-to-digital converter (ADC) are used. Techniques for improving their linearity and range resolution, respectively, are based on the design effort of the open-loop analog readout circuits like source followers and having a high demodulation frequency (or short pulse width) and high-full-well capacity [10,26]. A negative feedback technique in analog circuits and systems is known as an effective way for improving the nonlinearity, frequency response, and stability [27,28]. In the proposed design, the negative feedback technique is used at the time-domain for coarse and fine ToF measurements. The negative feedback used for coarse ToF measurements is based on finding zero of the ToF difference from the delay of gating pulses generated by a digital-to-time converter (DTC), effectively improving the linearity by maintaining a constant operating point of analog readout circuits. In fine ToF measurements, a first-order delta-sigma modulation (DSM) loop using time-domain feedback is used. The DSM loop using one-bit analog-to-digital converter (ADC) and one-bit digital-to-analog converter, which is popular for audio ADCs, has a self-contained property of low distortion and noise using oversampling signal processing [29,30,31]. It provides high-linearity and range resolution in ToF measurements if it is applied as the DSM using the time-domain feedback. A prototype chip for the concept proof is implemented, and the linearity and resolution are characterized. 

The rest of the paper is organized into Section 2 describing the principle of ToF measurements using time-domain feedback. Section 3 describes the circuit design, and Section 4 provides the results of the ToF measurement system implementation and measurements. Section 5 presents the concluding remarks.

## 2. ToF Measurement Using Time-Domain Feedback

**Figure 1:**
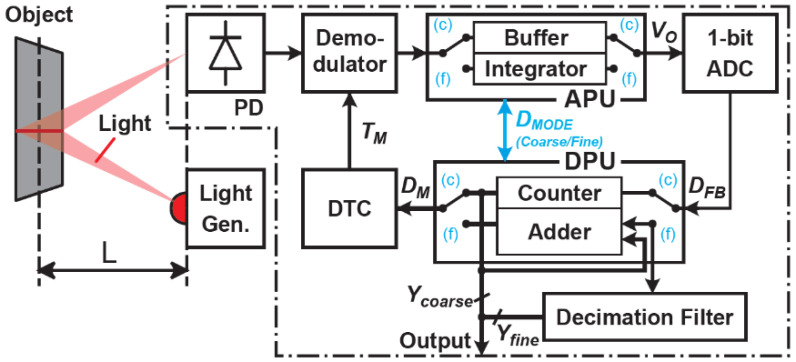
A system diagram of the proposed sensor.

Figure 1 shows a system diagram of the proposed sensor. The light pulse emitted from the light source is reflected onto the object and converted into an electrical signal by a photodiode (PD) and a demodulator. The time-domain negative feedback loop consists of a demodulator, analog processing unit (APU), 1-bit ADC, digital processing unit (DPU), and digital-to-time converter (DTC). This system measures the ToF with high linearity and resolution. To cover the wide range of ToF measurements while maintaining high-linearity and range resolution, the system in Figure 1 works with a coarse and fine measurement step. To realize the coarse and fine measurements with the time-domain negative feedback loop, each APU and DPU have two switchable functions. In the coarse measurement, a buffer amplifier and digital counter are used for the APU and DPU, respectively, working as an incremental ToF-to-digital conversion. In the fine measurement, an integrator and digital adder are used for the APU and DPU, respectively, working as a first-order DSM.

### 2.1. Incremental Time-to-Digital Conversion for Coarse ToF Measurements

**Figure 2:**
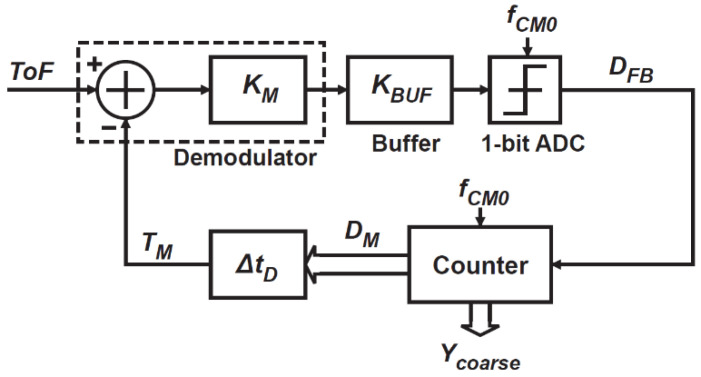
An equivalent block diagram for coarse time-of-flight (ToF) measurements.

Figure 2 shows an equivalent block diagram for coarse ToF measurements, consisting of a demodulator, buffer gain stage, 1-bit ADC, digital counter, and DTC. This system works as an incremental time-to-digital conversion (TDC). The demodulator senses the time position of ToF, and the demodulated charges are converted into a voltage signal at the buffer stage. With the time-domain feedback with DTC output, the voltage signal output of the demodulator with the buffer gain stage is expressed as

(1) $$V_{O}=K_{BUF}K_{M}(ToF-T_{M}),$$

where $K_{M}$ is the time-to-voltage conversion gain of the demodulator, $K_{BUF}$ is the voltage gain of the buffer, and $T_{M}$ is the DTC output in the time-domain. The 1-bit ADC quantizes the buffer output with a threshold of zero, and the increment code for the digital domain feedback, $D_{FB}$, is generated as

(2) $$D_{FB}=\{\begin{array}{cc} 0 & \left(V_{O}<0\right)\\ 1 & \left(V_{O}\geq 0\right) \end{array}.$$

The $D_{FB}$ is given to a counter where the number of 1’s is counted, and the counter output at the *n*-th step, $D_{M}(n)$, is expressed as

(3) $$D_{M}(n)=D_{M}(n-1)+D_{FB}.$$

**Figure 3:**
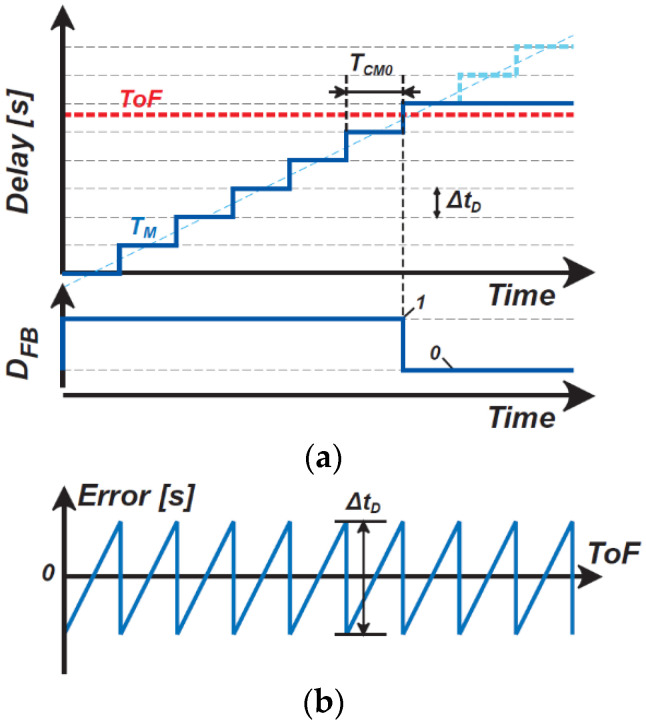
(**a**) Simplified conceptual operating waveform and (**b**) the quantization error of the ToF for coarse measurements.

The delay of DTC output at the *n*-th step, $T_{M}(n)$, which is the time-domain feedback to the demodulator, is given by

(4) $$T_{M}(n)=\Delta t_{D}\cdot D_{M}(n),$$

where $\Delta t_{D}$ is the conversion factor of DTC, which is equal to the DTC unit delay step. Figure 3 shows a simplified conceptual operating waveform and the ToF quantization error. To coarsely measure ToF using the incremental TDC, the initial value of the counter output, $D_{M}(0)$, is set to zero so that $T_{M}$ is initially set to zero and $T_{M}$ is gradually approximated step-by-step to ToF, expressed as a set of the above equations. While $D_{FB}$ is 1, the DTC delay is incremented. When $T_{M}$ becomes larger than ToF, $D_{FB}$ becomes 0 and the DTC output delay increment is stopped. When this happens at the $n_{C}$-th step, the counter output is expressed as

(5) $$D_{M}(n_{C})=n_{C}.$$

The counter output at the final step, $D_{M}(N_{C})$, is also $n_{C}$ because the counter output is not incremented after the $n_{C}$-th step, where $N_{C}$ is the maximum number of DTC delay steps. The DTC output at the final step, $T_{M}(N_{C})$, is given by $\Delta t_{D}\cdot n_{C}$. The difference between the ToF and $T_{M}(N_{C})$ is the error of the measured ToF because of the incremental TDC in the coarse ToF measurement mode. The error is maximal within the range of $-\Delta t_{D}/2$ to $\Delta t_{D}/2$ (Figure 3b). The maximum ToF measurement range is $T_{M,Max}$, given by

(6) $$T_{M,Max}=N_{C}\cdot \Delta t_{D}.$$

The output of the coarse ToF measurements, $Y_{coarse}$, is the final code stored in the counter as

(7) $$Y_{coarse}=n_{C}.$$

The time required for doing the coarse ToF measurement time, $T_{CM}$, is expressed as $T_{CM}=N_{C}\cdot T_{CM0}$ if the time required for doing one step for the gradual approximation is $T_{CM0}$.

### 2.2. DSM for Fine ToF Measurements

**Figure 4:**
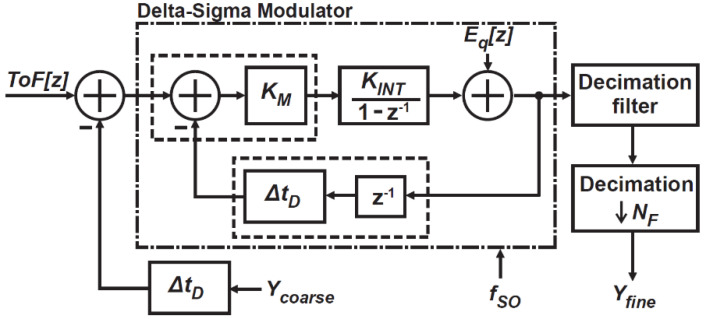
An equivalent block diagram for fine ToF measurements.

Figure 4 shows an equivalent block diagram for fine ToF measurements, employing a first-order delta-sigma modulation (DSM) with time-domain negative feedback. Though the use of higher-order DSMs has more efficient improvement of SNR to the oversampling ratio used, the first-order DSM is employed for an area-efficient column-wise implementation with a limited column pitch. In this fine ToF measurement mode, an integrator and adder are used for the APU and DPU, respectively. The adder adds coarse ToF measurement results stored in the counter, and the DSM finely measures the ToF difference from a coarse estimate of it, ΔTOF. From Figure 4, the DSM output *D_FB_*[*z*] is expressed as

(8) $$D_{FB}[z]=E_{q}(z)+\frac{K_{INT}K_{M}}{1-z^{-1}}D(z),$$

where $K_{INT}$ is the gain of the integrator, $E_{q}$ is the quantization noise of the 1-bit ADC, and D(z) is the difference of ΔTOF to the time domain feedback, expressed as

(9) $$D(z)=\Delta TOF(z)-\Delta t_{D}z^{-1}Y(z).$$

By substituting Equation (9) into (8), the overall system response in the z-domain for the DSM part of Figure 4 is expressed as

(10) $$D_{FB}[z]=\frac{K_{INT}K_{M}}{1-(1-K_{INT}K_{M}\Delta t_{D})z^{-1}}\Delta ToF[z]+\frac{1-z^{-1}}{1-(1-K_{INT}K_{M}\Delta t_{D})z^{-1}}E_{q}[z],$$

where $K_{INT}$ is the gain of the integrator, and $E_{q}$ is the quantization noise of the 1-bit ADC. The conventional first-order DSM using a 1-bit ADC shows that the loop gain of the modulator is unity if the modulator is stably operated [31]. Therefore, the condition $K_{INT}K_{M}\Delta t_{D}=1$ is met, and the equation is simplified as

(11) $$D_{FB}[z]=\frac{1}{\Delta t_{D}}\Delta ToF[z]+(1-z^{-1})E_{q}[z].$$

From Equation (11), transfer functions to signal and quantization noise are obtained. The signal transfer function, the relationship between $D_{FB}[z]$ and ΔTOF[z], is the same as the inverse of the DTC delay step. The noise transfer function (NTF), the relationship between $D_{FB}[z]$ and $E_{q}[z]$, is $1-z^{-1}$. In order to estimate the quantization noise using the DSM, it is useful to find the squared magnitude of NTF in the frequency domain [30] by setting $z=e^{j2\pi fT_{SO}}$, given by

(12) $${\left|NTF(e^{j2\pi fT_{SO}})\right|}^{2}={\left[2\sin(\pi f/f_{SO})\right]}^{2},$$

where f is the frequency, $T_{SO}$ is the period of the oversampling, and $f_{SO}(=1/T_{SO})$ is the oversampling frequency. 

**Figure 5:**
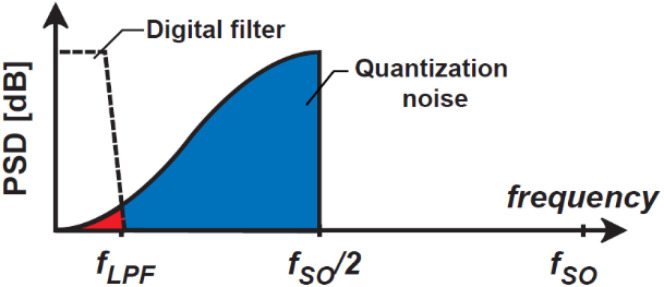
Noise transfer function of delta-sigma modulation.

Figure 5 shows the power spectrum density (PSD) due to the shaped quantization noise through the NTF given by Equation (10). The shaped quantization noise is reduced using a digital low-pass filter (LPF) with a cutoff frequency of $f_{LPF}$. Then, the ratio of the square root of the residual noise power (the red colored area) to the entire quantization noise (the sum of the red and blue colored areas) gives the increase of the effective number of bits (ENOB) in the fine TDC. With the oversampling ratio denoted by $N_{F}$ and given by $f_{SO}/2f_{LPF}$, the area ratio of red-colored to (red + blue)-colored is approximately $1/N_{F}{}^{3}$ [30,31]. Then the ENOB increase using an ideal LPF denoted by $\Delta ENOB_{i}$ is given by

(13) $$\Delta ENOB_{i}=\log_{2}N_{F}{}^{3/2}[\mathrm{bit}]=1.5\log_{2}N_{F}[\mathrm{bit}].$$

In this proposed technique using the first-order DSM, $\Delta ENOB_{i}=3$ [bit] is obtained for $N_{F}$ of 4. However, this is the case that an ideal low-pass filter, which has very steep cutoff, is used. In the actual implementation, a very simple counter that counts the number of 1’s in the bit-stream is used for the low-pass filtering. Because the counter does not have steep cutoff and its frequency response is like a moving average filter, the residual quantization noise power after low-pass filtering is $1/N_{F}{}^{2}$ of the entire quantization noise. The increase in the effective number of bits (denoted by $\Delta ENOB_{1}$) in this case is given by

(14) $$\Delta ENOB_{1}=\log_{2}N_{F}[\mathrm{bit}].$$

In the actual implementation, $N_{F}=64$ (or 66 for error reduction) and a counter-based low-pass filter is used, and the ENOB increase in the fine TDC implementation is 6 bits. From Equation (14), doubling the oversampling ratio $N_{F}$ can increase the ENOB by another one bit, while having the penalty of doubling the processing time of the fine ToF measurement. The filtered output of the DSM linearly responds to the ΔTOF, and a fine TDC for fine ToF measurement is realized. 

### 2.3. Coarse-to-Fine ToF Measurements

**Figure 6:**
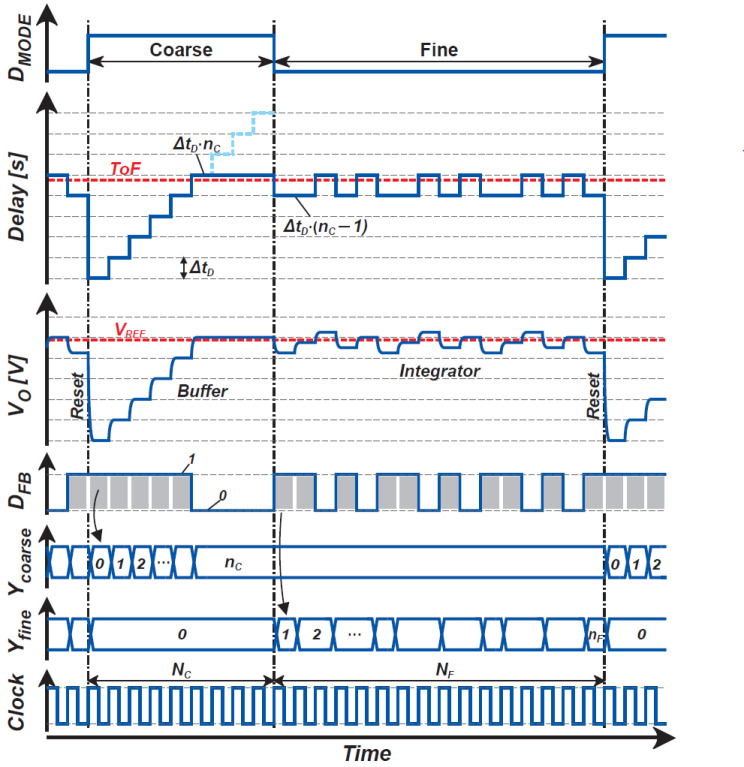
Conceptual operating waveform of the proposed system.

Figure 6 shows the conceptual operating waveforms of the proposed ToF measurement technique, including the incremental time-to-digital conversion and delta-sigma modulation for coarse and fine ToF measurements. After the coarse conversion, the incremental TDC output produces a coarse estimate of ToF, $\Delta t_{D}n_{C}$. In subsequent fine conversion using the DSM, DTC output produces a waveform of bit-stream and takes two states of $\Delta t_{D}n_{C}$ and $\Delta t_{D}(n_{C}-1)$. The DSM output is low-pass filtered and down-sampled for decimation, and the fine ToF measurement, or a fine TDC output, $Y_{fine}$ is produced. 

Figure 6 shows the filter output behavior when a digital counter (a first-order integrator) is used for the decimation filter. The filter output at the end of fine conversion is expressed as the number of 1’s of the DSM output, and if it is $n_{F}$ and the total sampling number is $N_{F}$, the output of fine ToF measurements $Y_{fine}$ is expressed as

(15) $$Y_{fine}=n_{F}/N_{F}.$$

$Y_{fine}$ takes the value from 0–1. The output using coarse-to-fine cascaded ToF measurements $Y_{TOF}$ is given by

(16) $$Y_{TOF}=Y_{coarse}+Y_{fine}-1$$

or

(17) $$Y_{TOF}=n_{C}+n_{F}/N_{F}-1.$$

If $N_{C}$ and $N_{F}$ are chosen as $2^{m_{C}}$ and $2^{m_{F}}$, this coarse-to-fine ToF measurement technique converts the ToF to a $\left(m_{C}+m_{F}\right)$-bit digital code as a result of incremental and DSM-based TDC techniques.

## 3. Circuit Design

### 3.1. Photo-Signal Demodulator

**Figure 7:**
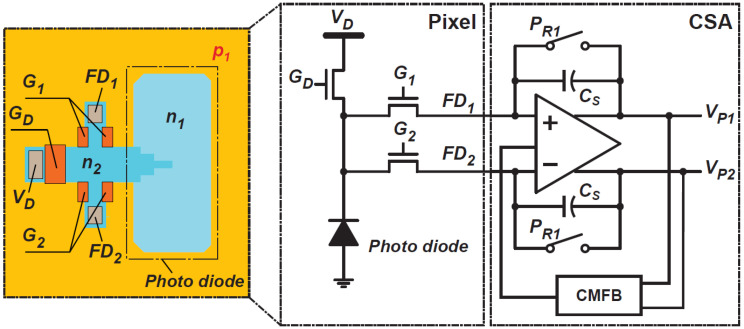
A circuit schematic of the photo-signal receiver and demodulator.

**Figure 8:**
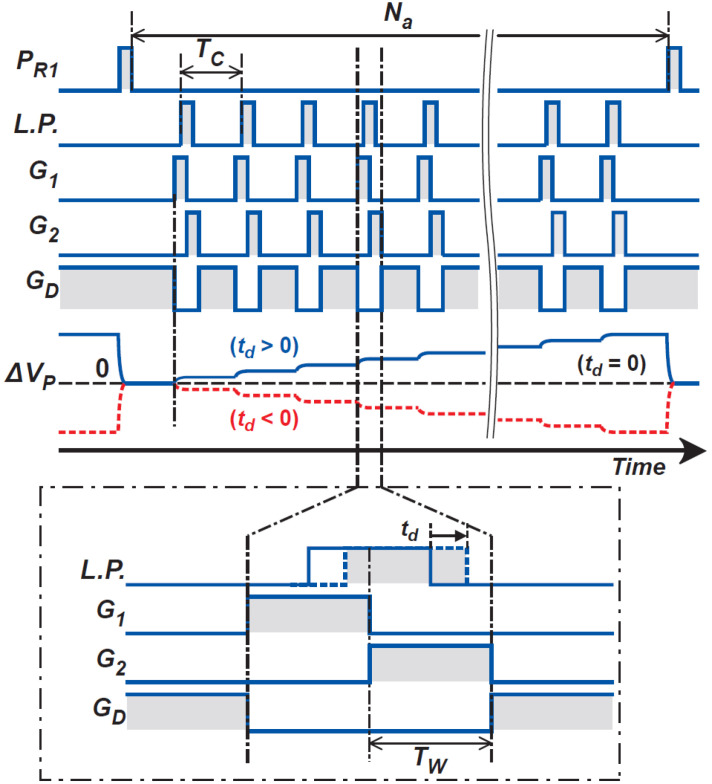
An operation-timing diagram for the photo-signal receiver and demodulator.

Figure 7 shows a circuit schematic of the photo-signal receiver and demodulator. Figure 8 shows the operation-timing diagram for the photo-signal receiver and demodulator. A PD in a pixel receives a periodic short-light pulse train and the generated electrons are transferred to a floating diffusion node (FD_1_), another floating diffusion node (FD_2_), or a drain, depending on the light pulse delay. The FD_1_ and FD_2_ are connected to the input of a fully differential charge-sensitive amplifier (CSA) to convert the modulated photo-charge to a differential voltage. The amplifier is a folded-cascode type with switched-capacitor common mode feedback (CMFB), having 77 dB gain and 53 MHz unit gain bandwidth at a phase margin of 76 degrees. The charge transfer control gates $G_{1}$, $G_{2}$, and $G_{D}$ demodulate the generated photo-signal. When photons move to the PD while $G_{1}$ is set to a high voltage level, the generated photoelectron is transferred to FD_1_. The photoelectron generated when the $G_{2}$ is set to a high level is transferred to FD_2_, and when the $G_{D}$ is set to a high level, they are drained by connecting the drain terminal to a voltage source $V_{D}$. For ToF measurements with high-resolution, the switching time for this demodulation must be sub-nanoseconds. The photoreceiver and demodulator are designed to perform this function, and the sub-nanosecond carrier response is based on the design reported in [21] where the photoreceiver is implemented with a lateral drift PD. The $G_{1}$ and $G_{2}$ are implemented with lateral electric field modulation (LEFM) gates, and the $G_{D}$ is implemented with a MOS transfer gate [22]. The charges $Q_{1}$ and $Q_{2}$ are transferred to FD1 and FD2, respectively, because of the operation of LEFM gates. Because of the operation of the CSA, the charges $Q_{1}$ and $Q_{2}$ are mainly transferred and accumulated in two feedback capacitors $C_{S}$ in the CSA. Using a common-mode feedback (CMFB) technique to control the average level of the fully differential output to be equal to a common level, $V_{COM}$, the output of the CSA, $V_{P1}$, and $V_{P2}$, is expressed as

(18) $$V_{P1}≅\frac{Q_{1}-Q_{2}}{2C_{S}}+V_{COM}\;\mathrm{and}$$

(19) $$V_{P2}≅\frac{Q_{2}-Q_{1}}{2C_{S}}+V_{COM}.$$

If the photo-generated charge occurs because of the signal light pulse for ToF measurements and ambient light, the $Q_{1}$ and $Q_{2}$ are expressed as

(20) $$Q_{1}≅Q_{S1}+Q_{B}\;\mathrm{and}$$

(21) $$Q_{2}≅Q_{S2}+Q_{B}$$

where $Q_{S1}$, $Q_{S2}$, and $Q_{B}$ are signal components of $Q_{1}$ and $Q_{2}$, respectively, and ambient light components. By using a fully differential amplifier with the CMFB, the differential output $\Delta V_{P}=V_{P1}-V_{P2}$ is given by

(22) $$\Delta V_{P}=V_{P1}-V_{P2}≅\frac{Q_{S1}-Q_{S2}}{C_{S}}.$$

The differential CSA output cancels the ambient light components and is proportional to the difference in the signal charge $Q_{S1}-Q_{S2}$. The charge-to-voltage conversion gain $G_{C}$ is given by

(23) $$G_{C}≅\frac{\Delta V_{P}}{Q_{S1}-Q_{S2}}=\frac{1}{C_{S}}.$$

Because a small number of photons are included in one short light pulse, the signal is intensified by receiving the light pulse periodically with many cycles. In Figure 8, the behavior of $\Delta V_{P}$ is shown for three cases of the relative delay of light pulse $t_{d}$. If $t_{d}$ equals 0 and the photoelectrons are equally shared in $Q_{S1}-Q_{S2}$, $\Delta V_{P}$ takes 0. If $t_{d}>0$, the CSA output signal gradually increases with positive polarity. If $t_{d}<0$, the CSA output gradually increases but with negative polarity. Therefore, the demodulator with the CSA can be used for measuring relative light pulse delays because of ToF. The CSA output is sampled by the next stage after the CSA output is sufficiently intensified, and the charge in $C_{S}$ is reset by turning on switch $P_{R1}$ to prepare for the next cycle.

### 3.2. Analog Processing Unit for Fixed-Gain Amplifier and Integrator

**Figure 9:**
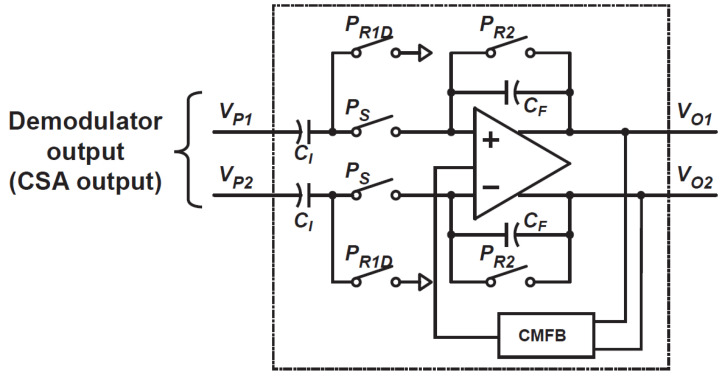
A circuit schematic of the analog processing unit (APU) for a fixed-gain amplifier and integrator.

Figure 9 shows a circuit schematic of the APU for a fixed-gain amplifier and integrator that are used for coarse and fine ToF measurements, respectively. In the coarse ToF measurement, the APU performs as a buffer to carry a photo-signal to the comparator at every feedback cycle. It is implemented using a fully differential switched-capacitor (SC) amplifier where the ratio of capacitance used for the input and feedback path sets the amplifier gain. It works with two operation phases. In the first phase, input capacitors $C_{I}$ sample the differential input signal by turning on switches $P_{R1D}$. During that time, the charge in feedback capacitors $C_{F}$ is reset by turning on switches $P_{R2}$. In the second phase, the switches $P_{R1D}$ and $P_{R2}$ are turned off, and switches $P_{S}$ are turned on. The charges sampled in $C_{I}$ is transferred to $C_{F}$ so that the capacitance ratio amplifies the input signal. The gain of the buffer, i.e., the gain of the differential output to its differential input $A_{I}\left(=\Delta V_{O}/\Delta V_{P}\right)$, is expressed as

(24) $$A_{I}=\frac{\Delta V_{O}}{\Delta V_{P}}=\frac{C_{I}}{C_{F}}.$$

In the fine ToF measurement, the APU performs as a fully differential SC integrator. In this operation mode, the switches $P_{R2}$ are turned on for resetting the charge in $C_{F}$ at the beginning. Input signal sampling by $C_{I}$ and $P_{R1D}$ and charge transfer to $C_{F}$ by switches $P_{S}$ are repeated to conduct SC integration. The final output after repeated SC integration with $N_{F}$ cycles, $\Delta V_{O}(N_{F})$, is given by

(25) $$\Delta V_{O}(N_{F})≅A_{I}\sum_{i=1}^{N_{F}}\Delta V_{P}(i),$$

where $\Delta V_{P}(i)$ is the *i*-th differential input.

### 3.3. DTC and Gating-Pulse Generator

**Figure 10:**
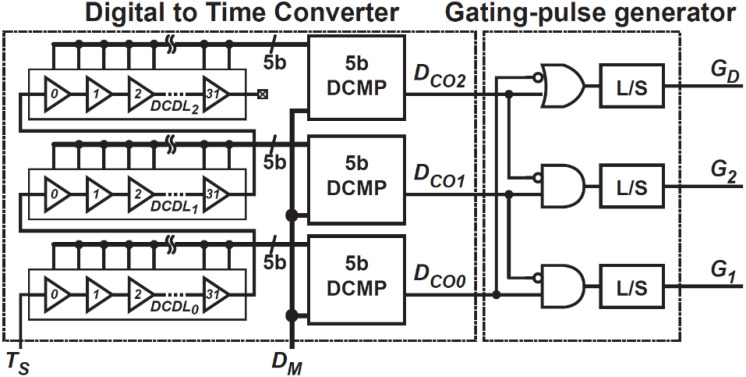
A circuit schematic of the digital-to-time converter and gating-pulse generator.

**Figure 11:**
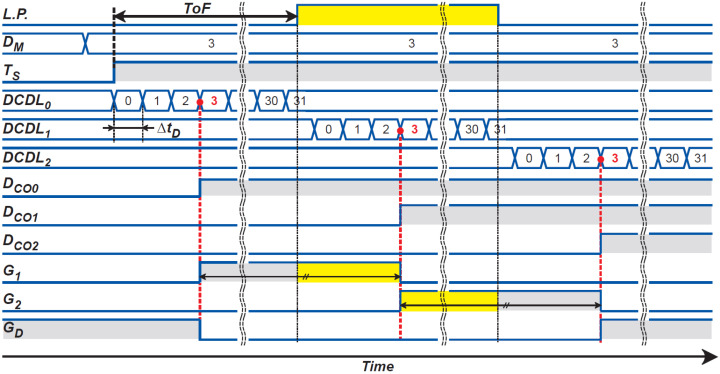
Conceptual operation waveform of the digital-to-time converter and gating-pulse generator.

Figure 10 shows the DTC design and gating-pulse generator for ToF measurements with time-domain feedback. A digitally controlled delay line (DCDL) generates a delay time of an integer multiple of a unit delay, $\Delta t_{D}$, and is configured with 32 stages for a 5-bit resolution of DTC. Figure 11 shows the operation waveform of DTC and gating-pulse generator. The DTC output generates the three-phase gating pulses, $G_{1}$, $G_{2}$, and $G_{D}$, by connecting 3 DCDLs, each of which has a 5-bit counter, in series, and the three 5-bit DCDL outputs are compared with $D_{M}$, which is the DPU output in Figure 1. The DCDL is triggered by a start pulse $T_{S}$. When $T_{S}$ is applied to the DCDL, three DCDLs start to generate a 5-bit binary counting code with the unit cycle time of $\Delta t_{D}$ (Figure 11). By comparing the three DCDL outputs with $D_{M}$, the gating-pulse generator generates a set of waveforms for $G_{1}$, $G_{2}$, and $G_{D}$, which is used for the photo-signal demodulator (Figure 7).

## 4. Implementation and Measurement

**Figure 12:**
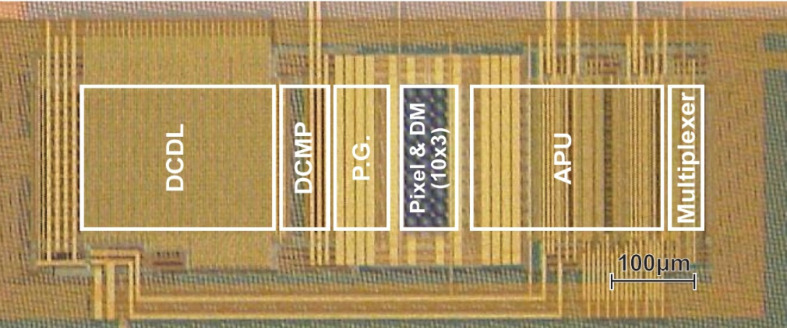
A photomicrograph of the prototype chip.

Figure 12 shows a photomicrograph of the prototype chip implemented in 0.11 μm (1P4M) CIS technology. The chip includes 10 × 3 pixels with the demodulator, 10 units of a set consisting of a DCDL, a 5-bit digital comparator (DCMP), a demodulation gate driver, and an APU. The pixel size is 16.8 × 16.8 μm^2^, the pitch of the readout circuit channel is 16.8 μm, and the chip core size is approximately 0.232 mm^2^. A multiplexer chooses the APU output to evaluate the characteristics of one channel. External components of a field-programmable gate array (FPGA), 1-bit comparator, light pulse generator, digital delay controller, and PC interface are used for implementing the proposed system in Figure 1.

**Figure 13:**
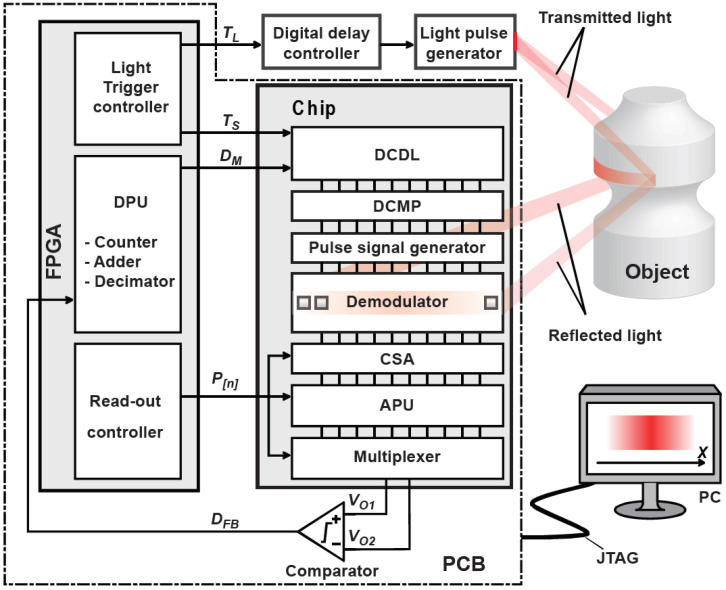
A measurement setup for demonstrating the proposed ToF measurement system.

Figure 13 shows the measurement setup for demonstrating the proposed ToF measurement system using the time-domain feedback technique. A specific channel multiplexed from the APU outputs of the implemented chip is connected to an external comparator, and the bit-stream signal output from the comparator is connected to the DPU implemented in the FPGA. The output of the DPU as a 5-bit digital code is fed back to the DCDL of the chip and controls the gating-pulse delay of the demodulator (DM). The FPGA also supplies the light source triggering pulse and operation clock signals for the CSA, APU, and multiplexer of the chip. The DPU implemented in the FPGA covers the function of a counter, a time-offset adder, a decimation filter, and a JTAG interface to PC. The digital output of the chip is finally transferred to the PC for characterizing the implemented ToF sensor chip.

**Figure 14:**
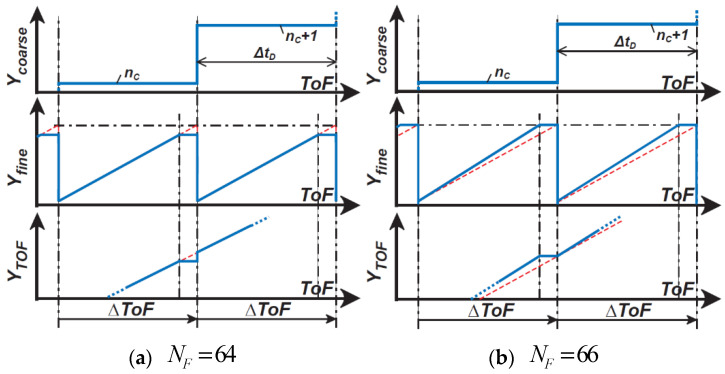
A conceptual operating waveform of $Y_{coarse}$, $Y_{fine}$, and the combined output $Y_{ToF}$ for $N_{F}$ of (**a**) 64 and (**b**) 66.

For demonstrating the ToF measurement using the proposed technique with time-domain feedback, a laser diode with a pulse width of 5 ns and a wavelength of 473 nm is used for the light source. The number of light pulses in each DSM oversampling period $N_{a}$ is 500. The oversampling frequency used for the DSM is 4.2 MHz. The total ToF measurement time, including both coarse and fine measurements, is 22.4 ms. The total DCDL delay is set to 7 ns with a unit step of 218 ps, and the maximum ToF measurement range is set to 105 cm. A digital delay generator delays and sweeps the trigger pulse of the light source, $T_{L}$, to characterize the response of the implemented ToF measurement system to the pseudo-distance. The maximum number of DTC delay steps $N_{C}$ is set to 32 for a 5-bit coarse conversion. The total sampling number $N_{F}$ is set to 64 and 66 for the fine DSM conversion. Figure 14 shows a conceptual operating waveform of $Y_{coarse}$, $Y_{fine}$, and the combined output $Y_{TOF}$ for $N_{F}$ of 64 (Figure 14a) and 66 (Figure 14b). Although $N_{F}$ of 64 exactly corresponds to the 6-bit fine ToF-to-digital conversion, it causes time-variant errors at specific codes when the coarse and fine conversion codes are connected. This is because a discontinuous step appears in the linearity curve at the boundary of the maximum ΔToF. At this dis-continuous point, a time-variant error occurs, and this extraordinarily worsens the range resolution (or depth noise). This can be avoided by increasing $N_{F}$ to be larger than 64. To address this issue,$N_{F}$ of 66, corresponding to the 6^+^ bits, is also evaluated for improving the depth resolution in Figure 14b. By increasing $N_{F}$ to be 66, the gain of $Y_{fine}$ to ToF is increased by a factor of 66/64 and the linearity curve of $Y_{fine}$ and the resulting $Y_{ToF}$ becomes as shown in Figure 14b. The discontinuous step in the linearity curve of $Y_{ToF}$ disappears, and the extraordinal depth noise at the discontinuous point is also reduced at the penalty of a little worse nonlinearity.

**Figure 15:**
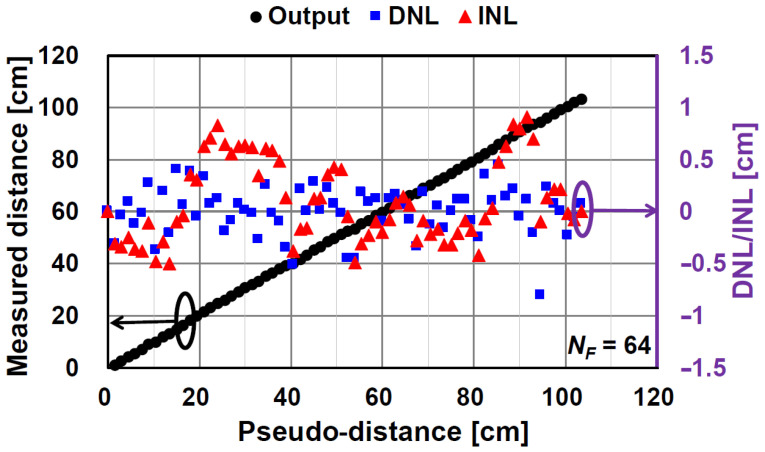
Measurement results of measured distance, differential nonlinearity (DNL), and integral nonlinearity (INL) of prototype chip ($N_{F}$ = 64).

**Figure 16:**
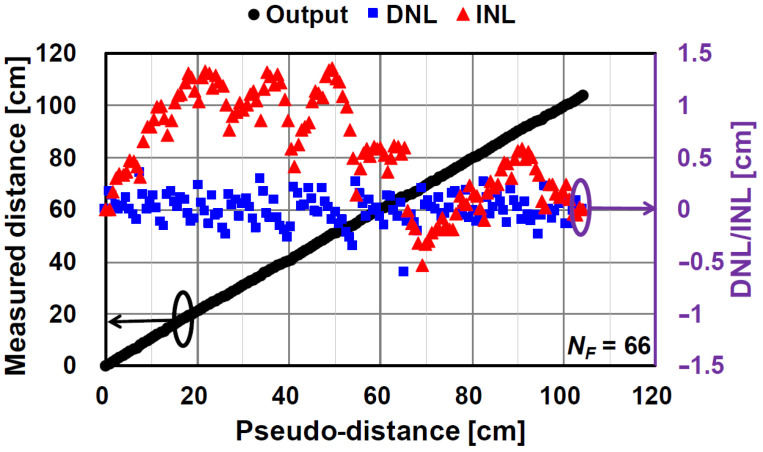
Measurement results of measured distance, DNL, and INL ($N_{F}$ = 66).

Figure 15 and Figure 16 show the results of the measured distance, differential nonlinearity (DNL), and integral nonlinearity (INL) for $N_{F}$ of 64 and $N_{F}$ of 66, respectively. A good linearity is obtained without any corrections for linearity improvements. The maximum INL for the case of $N_{F}$ of 64 is +0.9%/−0.47% (+0.94 cm/−0.49 cm) to the entire range of 105 cm. The maximum DNL for $N_{F}$ of 64 is +0.43%/−0.76% (+0.45 cm/−0.8 cm) to the entire range of 105 cm. For the case of $N_{F}$= 66, which is used for improving temporal errors, the maximum INL (+1.29%/−0.51% (+1.36 cm/−0.54 cm)) is a little worse than that for $N_{F}$ of 64 because the code resolution is not equal to 6 bits. However, the maximum DNL (+0.33%/−0.58% (+0.35 cm/−0.6 cm)) for the $N_{F}$ of 66 is even better than $N_{F}$ of 64 because the redundancy of the code ($N_{F}$ of 66 corresponds to a resolution of 6.044 bits) might improve the nonlinearity at the connecting points of the coarse and fine digital codes.

**Figure 17:**
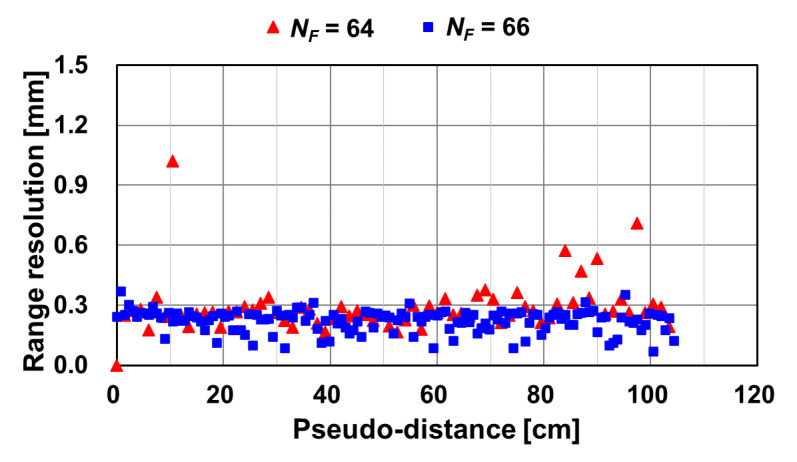
Measurement results of the depth resolution for $N_{F}=64$ and $N_{F}=66$.

Figure 17 shows the measurement results of the depth resolution to the pseudo-distance. Two cases of fine ToF measurement setting for $N_{F}$ of 64 and $N_{F}$ of 66 were compared. For $N_{F}$= 64, the range resolution of 0.27 mm (median) is good compared with other indirect ToF sensors in Table 1. However, at specific points, the range resolution is 3 times worse than its median, and the worst case is 1.02 mm. This extra-ordinal error is because of the temporal variation of nonlinearity at the connection points of the coarse and fine digital code. This problem is solved by increasing the effective number of bits for fine measurement using DSM. By using $N_{F}$ of 66, the range resolution (median) is improved from 0.27 mm to 0.24 mm, and the worst value is from 1.02 mm to 0.37 mm. Therefore, the code redundancy for fine ToF measurements might improve the temporal variation of nonlinearity at the connecting points of the coarse and fine digital codes, and the resulting depth resolution is entirely good.

The performance summary of the ToF sensor of this work and a comparison to other works are shown in Table 1. Because the implemented ToF sensor chip is a preliminary one having only 3(V) × 10(H) pixels and the design specifications are different from each other, this table is not intended for the overall chip performance comparison. With this table, however, the important feature of this work is clarified by the comparison of linearity and range resolution. In general, meeting both high linearity and high range resolution is difficult. A high range resolution needs a high-speed response of the pixel to use shorter light pulse modulation or higher modulation frequency, leading to distortions in the light-pulse waveform, photo-current waveform, and analog demodulation. The proposed technique using the time-domain negative feedback in this work is the best method for simultaneously meeting high linearity (+0.9%/−0.5%) and high range resolution (0.27 mm (median), 0.026% to full range (1.05 m)). Particularly for the comparison with Reference [26], which demonstrated sub-100 μm resolution, the advantage of this work is the large working range while attaining a good resolution. This feature comes from the proposed coarse-to-fine ToF measurement method, where the range is digitally extended by the incremental DTC and resolved finely with the short pulse width of 5 ns and oversampled noise suppression in the delta-sigma modulation.

Table 2 shows comparison of ToF sensor architectures including this work and two other typical ToF sensor architectures. The indirect ToF sensors using CW (Continuous Wave) and SP (Short Pulse) modulation uses simple analog readout circuits like a source follower. Because of this, it is suitable for implementing large area-array (2D pixel array)-type ToF image sensors. However, the analog readout types have issues of the nonlinearity and stability due to the analog elements like a photo-demodulator and amplifier. Because the role of the indirect ToF pixel is just the demodulation of modulated photo signals, the processing for range calculation and ambient light canceling have to be done by post-processing at an external system. In contrast to the indirect ToF sensors using analog readout circuits, the readout circuits per pixel of the direct ToF sensor using the SPAD and this work are based on digital-processing techniques. Though the readout and processing circuits are relatively complicated when compared with those of the indirect ToF sensors, there is a distinct advantage in its principle for having a high accuracy (high linearity) because of the use of the digitally regulated time reference. The direct ToF sensor using time stamping and the digital ToF sensor using the time-domain feedback technique (this work) use a time-to-digital converter and digital-to-time converter, respectively, for measuring the ToF, and the accuracy (linearity) is dominated by these digital elements, whose accuracy and stability can be much better than the analog counterparts. In these direct ToF sensors, the range calculation and the ambient-light cancellation are self-contained because the digital output is just the ToF itself. The biggest advantage of the direct ToF sensor using SPAD lies in its single-photon sensitivity, and therefore the SPAD-based direct ToF sensors are becoming a key technology for long-distance (>50 m) LiDAR applications. On the other hand, its photon-based processing may cause a difficulty in treating many photons, and the dynamic range is limited by the processing speed of the time-stamping of a photon. Therefore, the SPAD-based direct ToF sensors are not always the best solution for distance measurements for a few meters and with strong signals. The per-pixel readout circuits of the digital ToF sensor using the time-domain feedback are more complicated than that of the indirect ToF sensors but less complicated than that of the SPAD-based direct ToF sensors. The ambient light canceling is done by an analog fully differential charge-sensitive amplifier (CSA) during the demodulation process. While the dynamic range is limited by the full well capacity (FWC) of the fully differential CSA, the use of oversampling done in the delta-sigma modulation is effective for extending the dynamic range and reducing the influence of shot noise, because the integration of the sampled CSA outputs in the integrator has an effect of improving the shot-noise-limited signal-to-noise ratio. As a result, high range resolution (0.27 mm @ 0–1.05 m) is attained in this work. Because of the complicated analog and digital circuits per pixel, the proposed circuits architecture is suitable only for linear-array (1D) ToF image sensors.

## 5. Conclusions

A high-linearity high-depth resolution ToF linear-array digital image sensor using time-domain feedback that works as incremental gating-pulse delay feedback and DSM for coarse and fine ToF measurements, respectively, has been presented. The coarse ToF measurement uses time-domain feedback using the gating-pulse delay generated by a 5-bit DTC. The fine ToF measurement uses a first-order DSM for time-domain bit-stream feedback and oversampling signal processing for attaining high piecewise linearity and low depth noise (high depth resolution).

For a demonstration of the proposed system, a prototype sensor chip was implemented with a 0.11 μm (1P4M) CIS process. The chip contains a DTC, pulse generator, demodulation pixels, APU, and multiplexer. The other necessary functional blocks, such as a comparator, the DPU, timing controller, light source, and digital delay generator, are implemented using discrete components or an FPGA. The ToF measurement system produces an 11-bit fully digital output with a measurement cycle time of 22 ms. The prototype ToF system observed good linearity of +0.9%/−0.47%. An excellent range resolution of 0.24 mm (best case) for the range of 0–1.05 m is demonstrated. The residual nonlinearity is predominantly because of the deviation of the delay elements in the implemented DTC, which will be improved by introducing a precise DTC.

## Figures and Tables

**Table 1:** Performance summary and comparison.

	This Work	JSSC’19 [26]	JSSC‘19 [15]	JEDC’15 [22]	JSSC’15 [10]	Sensors’20 [23]
Array Type	Linear (1D)	Linear (1D)	Area (2D)	Area (2D)	Area (2D)	Area (2D)
Process	0.11 μm FSI	0.11 μm FSI	67 nm BSI	0.11 μm FSI	0.13 μm FSI	0.11 μm FSI
ToF Type	Indirect SP	Indirect SP	Indirect CW	Indirect SP	Indirect CW	Indirect SP
Readout Architecture	Time-Domain Feedback	Analog + Column ADC	Analog + Column ADC	Analog + Column ADC	Analog + Column ADC	Analog + Column ADC
Pixel Pitch	16.8 μm	22.4 μm	7 μm	16.8 μm	10 μm	22.4 μm
Pixel Number	3 (V) × 10 (H)	8(V) × 257(H)	480(V) × 640(H)	240(V) × 413(H)	424(V) × 512(H)	128(V) × 134(H)
Pixel Type	2-Tap	3-Tap	4-Tap	3-Tap	2-Tap	8-Tap
Light Wavelength	473 nm	473 nm	940 nm	870 nm	860 nm	850 nm
Modulation Frequency (Pulse Width)	5 ns	<80 ps	100 MHz	13 ns	10–130 MHz	6 ns
Range	(pseudo) 0–1.05 m	0–25 mm	0.4–4 m	0.8–1.8 m	0.8–4.2 m	1–6.4 m
Nonlinearity	+0.9%/−0.5% (0.94 cm)	<1% (<0.25 mm)	-	1.9% (<2 cm)	-	0.67% (<4 cm)
Resolution (rms) (% to full range)	0.27 mm (0.026%)	64 μm (0.256%)	<0.62%	7 mm (0.7%)	15 mm @ 3.5 m (0.441%)	8 mm (0.148%)

**Table 2:** Comparison of ToF Sensor Architectures.

	Indirect ToF Using CW or SP Modulation	Direct ToF Using SPAD	This Work (Digital ToF with Time-Domain Feedback)
Readout circuits per pixel	- Analog - Simple	- Digital (Time Stamping) - Complicated	- Digital (Time-Domain Feedback) - Complicated
What limits the linearity	- Analog Elements (Demodulator & Amplifier)	- Digital Elements (Time-to-Digital Converter)	- Digital Elements (Digital-to-Time Converter)
Ambient light canceling	- Post Processing	- On chip - Sophisticated but Complicated - Digital Processing	- On chip - Simple - Analog Processing
Range calculation	- Post Processing	- Self-Contained	- Self-Contained
What determines the dynamic range	- FWC in FDA	- Photon-Based Processing - Pile-up of Photon Signals	- FWC in CSA - Oversampling Ratio
Suitability to pixel-array type	- Linear Pixel-Array (1D) - Area Pixel-Array (2D)	- Linear Pixel-Array (1D) - Area Pixel-Array (2D)	- Linear Pixel-Array (1D)
Suitability to weak-signal application	- Fair	- Good	- Fair
Range resolution (Precision)	0.148% (8 mm) @ (1–6.4m) [23]	1.4mm @ (2m–50m) [5]	0.27mm @ (0–1.05m, Pseudo Distance)

## Data Availability

Data available in a publicly accessible repository.
